# Populus biomass protein-protein interactions and their functions

**DOI:** 10.1186/1753-6561-5-S7-O38

**Published:** 2011-09-13

**Authors:** Xiaoyan Jia, Mingzhe Zhao, Chengsong Zhao, Xiaoyan Sheng, Allan Dickerman, Eric Beers, Amy Brunner

**Affiliations:** 1 Department of Forest Resources and Environmental Conservation, Virginia Tech, Blacksburg, VA 24060, USA; 2 Department of Horticulture, Virginia Tech, Blacksburg, VA 24060, USA; 3 Virginia Bioinformatics Institute, Virginia Tech, Blacksburg, VA 24060, USA

## 

Proteins are molecular machines that play roles in almost all biological activities through interactions with other molecules such as carbohydrates, lipids, nucleic acids and other proteins. We are mapping protein-protein interactions relevant to woody biomass production by focusing on proteins co-expressed in poplar secondary xylem. In addition to revealing novel regulatory mechanisms important to woody biomass production, mapping the poplar protein-protein interactome will provide fundamental information relevant to xylem differentiation and secondary growth. We have cloned a portion of the poplar biomass ORFeome, specifically 374 ORFs that are upregulated in xylem versus phloem, for use in protein-protein interaction research. Here we summarize the techniques we are using to discover and study protein-protein interactions and results to date.

1. Yeast two-hybrid (Y2H) binary assays: 108,205 Y2H binary assays involving over 300 biomass ORFeome members have been performed and 11 interaction pairs identified. The proportional yield from our binary screen is similar to that represented by the current preliminary binary screen data from the Arabidopsis interactome project.

2. Y2H cDNA library screening: We have used 40 bait proteins to screen our poplar xylem cDNA prey library, and a total of 60 biomass ORFeome members comprising putative regulators of lignocellulose synthesis will be screened in 2011. For the 26 ORFs that are completely through the library screen, we have identified 44 unique interacting sequences. Thus far, the proportional yield of interactors for proteins catalyzing metabolic reactions (such as cellulose synthase, PB138) is much lower than that for regulatory proteins (such as NIMA kinase, PB223). Results from these Y2H screens are available at our project website (http://xylome.vbi.vt.edu/index.html). Additional website information includes a project overview, project objectives, progress to date, sequences for all ORFs and primers, clone availability, and Y2H protocols.

3. Confirmation of selected protein-protein interactions identified by Y2H screens: Selected Y2H interactions are being confirmed by independent methods including bimolecular fluorescence complementation (BiFC) and co-immunoprecipitation/affinity purification using plant transient or stable expression systems. We have developed improved vectors and protocols for reducing background fluorescence during BiFC experiments.

4. Functional analysis: We are characterizing the functions of selected interacting pairs in both poplar and Arabidopsis by ectopically expressing or suppressing genes singly and in combination. For ectopic and/or over-expression experiments we are using *35S*, *SUC2* (phloem specific) and *XCP2* (xylem specific) promoters. Co-expression of *35S::PB15* and *35S::PB129* in Arabidopsis resulted in expanded interfascicular regions containing enlarged fibers compared to fibers in wild-type plant interfascicular regions of the inflorescence stem. Importantly, this phenotype was not observed in transgenics overexpressing just one of these genes, showing the potential of novel interactome data to be translated into alteration of wood phenotypes.Arabidopsis mutants carrying T-DNA insertions within Arabidopsis orthologs of *PB15* and *PB129* are also being evaluated, and *Populus* RNA interference (RNAi) lines have been generated to study the effects of silencing *PB15* and/or *PB129* and their paralogs.

5. Transgenic poplar field trial: We prepared transgenic poplar overexpressing 11 biomass genes as tandem affinity purification-(TAPa)-tagged fusions and established these in a replicated field trial, which will be evaluated for overexpression phenotypes and serve as a source of xylem for identification of co-purified proteins.

